# The impact of enhanced screening for carbapenemase-producing Enterobacterales in an acute care hospital in South Korea

**DOI:** 10.1186/s13756-023-01270-8

**Published:** 2023-07-03

**Authors:** Sun Hee Park, Yunmi Yi, Woosuck Suh, Seul Ki Ji, Eunhee Han, Soyoung Shin

**Affiliations:** 1 Infection Prevention and Control Unit, Daejeon St. Mary’s Hospital, The Catholic University of Korea, Daejeon, Republic of Korea; 2 Division of Infectious Diseases, Department of Internal Medicine, College of Medicine, The Catholic University of Korea, Seoul, Republic of Korea; 3 Vaccine Bio Research Institute, College of Medicine, The Catholic University of Korea, Seoul, Republic of Korea; 4 Department of Pediatrics, College of Medicine, The Catholic University of Korea, Seoul, Republic of Korea; 5 Department of Laboratory Medicine, College of Medicine, The Catholic University of Korea, Seoul, Republic of Korea; 6 The Catholic University of Korea, Eunpyeong St. Mary’s Hospital, 93-19 Jingwan-dong, Eunpyeong-gu, Seoul, Republic of Korea

**Keywords:** Carbapenemase-producing Enterobacterales, Active screening, Periodic screening, Infection prevention and control

## Abstract

**Background:**

Carbapenemase-producing Enterobacterales (CPE) poses a significant challenge to infection control in healthcare settings. Active screening is recommended to prevent intra-hospital CPE transmission.

**Methods:**

CPE screening was initiated at a 660-bed hospital in South Korea in September 2018, targeting patients previously colonized/infected or admitted to outside healthcare facilities (HCFs) within 1 month. Universal intensive care unit (ICU) screening was performed at the time of admission. After a hospital-wide CPE outbreak in July-September 2019, the screening program was enhanced by extending the indications (admission to any HCF within 6 months, receipt of hemodialysis) with weekly screening of ICU patients. The initial screening method was changed from screening cultures to the Xpert Carba-R assay. The impact was assessed by comparing the CPE incidence per 1000 admissions before (phase 1, September 2018-August 2019) and after instituting the enhanced screening program (phase 2, September 2019-December 2020).

**Results:**

A total of 13,962 (2,149 and 11,813 in each phase) were screened as indicated, among 49,490 inpatients, and monthly screening compliance increased from 18.3 to 93.5%. Compared to phase 1, the incidence of screening positive patients increased from 1.2 to 2.3 per 1,000 admissions (*P* = 0.005) during phase 2. The incidence of newly detected CPE patients was similar (3.1 vs. 3.4, *P* = 0.613) between two phases, but the incidence of hospital-onset CPE patients decreased (1.9 vs. 1.1, *P* = 0.018). A significant decrease was observed (0.5 to 0.1, *P* = 0.014) in the incidence of patients who first confirmed CPE positive through clinical cultures without a preceding positive screening. Compared to phase 1, the median exposure duration and number of CPE contacts were also markedly reduced in phase 2: 10.8 days vs. 1 day (*P* < 0.001) and 11 contacts vs. 1 contact (*P* < 0.001), respectively. During phase 2, 42 additional patients were identified by extending the admission screening indications (n = 30) and weekly in-ICU screening (n = 12).

**Conclusions:**

The enhanced screening program enabled us to identify previously unrecognized CPE patients in a rapid manner and curtailed a hospital-wide CPE outbreak. As CPE prevalence increases, risk factors for CPE colonization can broaden, and hospital prevention strategies should be tailored to the changing local CPE epidemiology.

**Supplementary Information:**

The online version contains supplementary material available at 10.1186/s13756-023-01270-8.

## Background

Carbapenemase-producing Enterobacterales (CPE) poses a significant threat to global health, and the prevalence of CPE has increased worldwide. In South Korea, the prevalence of CPE has steadily increased since 2010, after the first identification of imported case [1], and mandatory notification of carbapenem-resistant Enterobacterales (CRE) infection/colonization to the Korea Disease Control and Prevention Agency (KDCA) was initiated in June 2017. Since then, the number of reported CRE cases has increased substantially from 5,717 to 2017 to 23,311 in 2021, with CPE accounting for 63.3% of the CRE cases in 2021 [1, 2].

Early detection and isolation of patients infected or colonized with CPE are the main infection control strategies to contain the spread of CPE in healthcare settings. For this purpose, many countries have developed national infection control guidelines based on the active screening of patients at high risk of CPE colonization or hospitalized patients in high-risk units [3–8]. However, it is the responsibility of individual facilities to make decisions regarding whom to screen and how to screen. This has led to variability in CPE screening strategies among hospitals [9]. The KDCA guidance recommends screening of patients who are at risk of CPE colonization at the time of admission; suggested risk factors include a history of contact with a CPE patient or admission to healthcare facilities (HCFs) where CPE outbreaks have occurred, or previous CPE colonization or infection. However, in the KDCA guidance, the duration of risk is not specified, and CPE screening policy primarily depends on the healthcare facilities’ decisions [10]. In South Korea, most hospitals have multi-occupancy rooms with shared bathrooms and open-bay design intensive care units (ICUs). In hospital settings, where patient isolation is difficult owing to the shortage of single rooms, active surveillance is often discouraged. Rapid detection is essential for effectively reducing the duration of exposure to CPE when preemptive isolation is difficult. However, conventional and commonly-used screening methods, such as culturing samples on selective media, require 24–48 h for growth, and molecular methods are required for confirmation [4]. Therefore, containment of CPE is a challenge in Korean healthcare facilities.

In 2017, the first case of CPE was identified at Daejeon St Mary’s Hospital in Daejeon, South Korea. In September 2018, we launched an admission CPE screening program targeting patients at risk of CPE colonization with limited indications, including previous CPE colonization/infection, previous admission to outside HCFs within the past month, transfer from outside HCFs, and ICU admission. Screening was performed using two consecutive rectal swabs sampled on chromogenic agar. Between July and September 2019, a CPE outbreak occurred in several wards. Thus, as of mid-August 2019, an enhanced screening program was implemented by broadening the screening indications and updating the screening protocol to include a rapid polymerase chain reaction (PCR) method, the Xpert-Carba-R assay, with culture-based methods.

This study aimed to evaluate the impact of changes in CPE screening strategies on the spread of CPE and development of clinical infections. We explored the trend of CPE colonization/infection over time and compared CPE incidence in screening cultures and clinical cultures before and after the introduction of the enhanced screening program. We also investigated the effect of the enhanced screening program on intra-hospital transmission of CPE.

## Methods

### Ethics

This study was approved by the Institutional Review Board at the Catholic University of Korea, Daejeon St. Mary’s Hospital, and the need for informed consent was waived (DC21ENSI0040).

### Hospital settings

This retrospective observational cohort study was conducted at the Catholic University of Korea, Daejeon St. Mary’s Hospital from April 2017 to December 2020. The study population included all hospitalized patients aged ≥ 18 years and those who stayed in the ICU for > 24 h. Our hospital is a 660-bed, university-affiliated secondary care hospital in Daejeon, South Korea. Daejeon has a population of 1.5 million people, and this hospital has an average of 24,300 admissions per year. Hospital general wards are composed of 95% multi-occupancy rooms with shared bathrooms, 5% en-suite single rooms, and four airborne infection isolation rooms. There are two ICUs (medical and surgical) that have an open bay design and two isolation rooms per ICU. Each ICU accommodates 18 patients.

### Microbiology tests

For screening cultures, ChromID CARBA agar (bioMérieux, France) was used to culture rectal swab specimens. The Xpert Carba-R assay was used to detect carbapenemases. CPE positivity was defined as both the Xpert Carba-R assay and screening culture being positive. Carbapenemase genes were further subtyped using PCR and sequencing as previously described [11]. When the Xpert Carba-R assay was negative but the cultures were positive, the isolates were considered CRE. Species identification was performed using matrix-assisted laser desorption/ionization time-of-flight mass spectrometry (Bruker, Daltonics, Germany), and antimicrobial susceptibilities were determined according to Clinical and Laboratory Standards Institute guidelines using the MicroScan WalkAway 96 Plus system and Neg Combo Panel Type 72 (Beckman Coulter, Brea, California).

### CPE screening programs and patient management

CPE screening programs and patient management strategies have changed over time, as shown in Table 1. In brief, CPE screening was not performed during phase 0 (April 2017-August 2018). During phase 1 (September 2018-August 2019), we performed the screening program with limited indications using only culture-based screening. If the screening culture was positive, the patients were isolated in a single room. During phase 2 (September 2019-December 2020), the indications for admission screening were expanded, the Xpert Carba-R assay was used for initial testing, along with culture-based methods (Table 1). If the Xpert Carba-R test was positive, the patients were isolated until two consecutives screening cultures were negative. If CPE colonization was confirmed, isolation was continued. During phase2, in addition to universal ICU admission screening, ICU patients were screened weekly during their stay, using cultures. Throughout the study period, preemptive isolation was performed only for patients with documented history of previous CPE colonization/infection. Previous CPE colonization/infection was verified through flags in electronic medical records and microbiology results at this hospital, and through inter-facility communication.

**Table 1:** Changes in carbapenemase-producing Enterobacterales control strategy during the entire study period (2017–2020)

	Phase 0	Phase 1	Phase 2
General ward Admission screening	None	Colonization/infection with CPE within 6 months prior to admission	Colonization/infection with CPE within 6 months prior to admission
		Previous admission to outside healthcare facility^1^ within the past 1 month	Previous admission to any healthcare facility^1^ within the past 6 months
		Transfer from long-term care facilities or acute care hospitals	Transfer from long-term care facilities, acute care hospitals, rehabilitation centers or nursing homes
			Receipt of hemodialysis
ICU Admission screening	None	Universal screening for all patients admitted to ICU	Universal screening for all patients admitted to ICU
ICU Periodic screening	None	None	Weekly screening during the ICU stay
Screening method	None	Two consecutive rectal swab cultures on chromogenic agar with a 24-hour interval, followed by Xpert Carba R assay if cultures are positive	Xpert Carba R assay, followed by two consecutive rectal swab cultures on ChromID CARBA agar with a 24-hour interval
Patient Isolation^2^	Patient isolation once clinical cultures reported as positive	Patient isolation once cultures reported as positive	Patient isolation once Xpert Carba R assay reported as positive until the following two rectal swab cultures reported negative. Isolation continued if cultures reported as positive
Patient De-isolation	De-isolation when 3 consecutive cultures with 1 week interval reported negative	De-isolation when 3 consecutive cultures with 1 week interval reported negative	De-isolation when 3 consecutive cultures with 1 week interval reported negative and at least 6 months have elapsed since the first negative conversion

Abbreviation: ICU, intensive care unit

Footnote: ^1^Acute care hospitals, long-term care hospitals, nursing homes and rehabilitation hospitals were included. ^2^Preemptive isolation was performed only for patients with previous CPE colonization/infection

Management of CPE positive patients was as follows. If CPE was confirmed, patients continued to be isolated in a single room until discharge. In case of a shortage of single rooms, CPE patients were cohorted in multi-occupancy rooms according to carbapenemase gene and organism type. Other infection prevention measures included (1) the signage on isolation room doors and flags in electronic medical records, (2) the use of personal protective equipment (PPE) including single-use gloves and gowns whenever entering the room, (3) the use of disposable or dedicated patient care equipment, (4) cohorting of nursing and cleaning staff in the event of an outbreak, and (5) environmental cleaning (twice daily) of isolation rooms and terminal cleaning with hypochlorite solution after patient discharge.

Contact management was similar during the study period. When any new CPE patients were identified, all contacts were traced and screened using cultures. The Xpert-Carba-R assay was also used for contact screening of ICU patients during phase 2. CPE contacts were cohorted until they were cleared after obtaining two negative swabs > 24 h apart.

### Staff education on CPE management

Since 2017, the hospital’s healthcare workers (HCWs) have received regular education and updates on CPE management according to the changes in the hospital policies. The CPE screening program was communicated to the staff before its launch in September 2018. In response to the outbreak, comprehensive training was conducted in in-person meetings, with active involvement of the hospital leadership, to educate all relevant HCWs in CPE management. Education programs were customized to suit the specific requirements and roles of each department in containing the outbreak. The hospital-wide campaign was initiated and continued to reinforce the enhanced CPE screening program. The performance of hand hygiene, implementation of contact precaution measures, and adherence to CPE screening protocols were monitored and communicated to both HCWs and leadership.

### Definitions

Compliance with admission screening was calculated by dividing the number of patients who were screened ≤ 48 h after admission by the number of patients who were indicated for screening. Compliance with weekly ICU screening was calculated by dividing the number of patients screened weekly by the number of patients who stayed in the ICU for ≥ 7 days.

The CPE exposure duration was defined as the period from either the admission date or the date when the most recent rectal swab culture was negative during admission to the date of initiation of contact isolation. Patients were considered CPE contacts if they shared a room with a CPE patient during the exposure period.

CPE patients were epidemiologically categorized based on timing of specimen sampled and the presence of risk factors as follows: (1) community-associated (CA): infection detected ≤ 2 days after admission to this hospital and with no known exposure to healthcare facilities; (2) community-onset, healthcare associated (CO-HA): infection detected ≤ 2 days after admission, with a history of hospitalization, long-term care facility (LTCF) residence, or hemodialysis within the previous 6 months; (3) Healthcare-onset, outside healthcare facilities (HO-OHCF): infection detected ≤ 2 days from admission after transfer from outside healthcare facilities; (4) healthcare-onset (HO): infection detected > 2 days after admission to this hospital.

CPE isolates obtained from a single patient were considered duplicates if they were the same species/carbapenemase combination, regardless of the source of culture specimens. Otherwise, CPE isolates without such concordance were considered as non-duplicates. CPE-positive clinical cultures included CPE isolates which were detected in clinical specimens either before or after screening cultures, or in cases where CPE screening was not conducted.

### Data collection and analysis

We collected data on patient demographics and the risk factors described above, the timing of specimen sampling and reporting, exposure duration, number of contacts, and CPE screening results. To assess the impact of the enhanced screening program, the incidence of CPE colonization/infection per 1,000 admissions was calculated during phases 1 and 2 and compared using Poisson regression. Given the outbreak period coincided with parts of both phase 1 and phase2, further analysis was conducted by dividing the study period into three periods: pre-outbreak (September 2018-June 2019), outbreak (July-September 2019), and post-outbreak (October 2019-December 2020), in order to assess the impact of the enhanced screening program in an endemic setting. The Chi-squared test or Fisher’s exact test was used to compare categorical variables. The Student’s *t*-test or Wilcoxon rank-sum test was used to compare continuous variables. Risk factors for positive admission screening were evaluated using log-binomial regression. Multivariate analyses were conducted, adjusting for age, sex and patient characteristics which were found significant in the bivariate analysis (*P* < 0.1). All analyses were performed using Stata (version 17.0; StataCorp, LP, College Station, TX, US). *P* < 0.05 was considered statistically significant.

## Results

### Patient demographics and compliance to the enhanced program

**Fig. 1:**
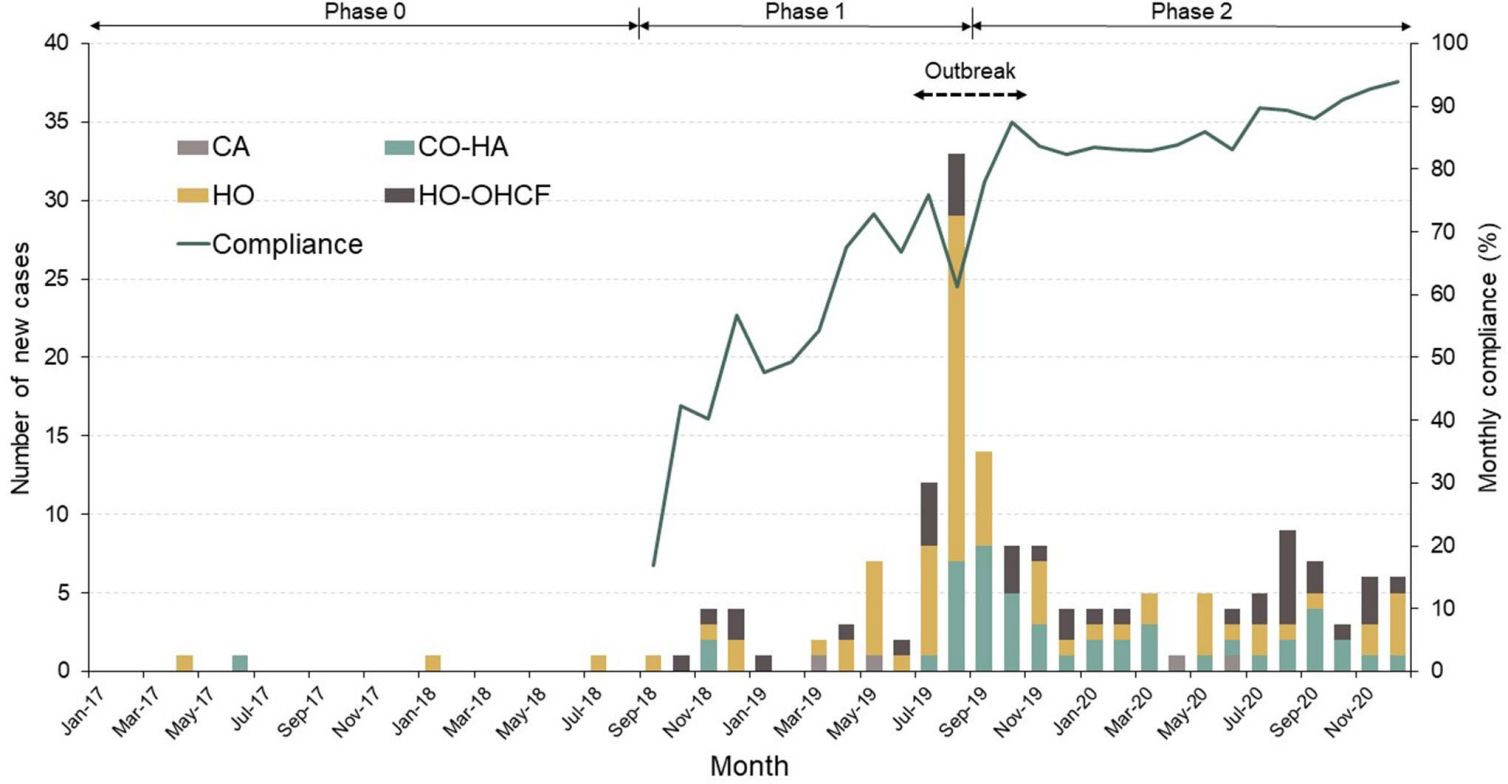
Trend in newly detected CPE cases according to epidemiological category and compliance to the screening program (2017–2020) Abbreviation: CA, community-associated; CO-HA, community-onset, healthcare-associated; CPE, carbapenemase-producing Enterobacterales; HO, hospital-onset; HO-OHCF, hospital-onset at outside healthcare facilities (transferred from outside healthcare facilities).

During the study period, 80,348 patients (30,858, 22,228, and 27,262 during phases 0, 1, and 2, respectively) were admitted to this hospital, and 7,413 episodes of ICU admission were identified among 6,555 patients. Over time, there was a steady increase in the proportion of patients who had been hospitalized in acute care hospitals (ACHs) or resided in LTCFs during the previous 6 months and who were transferred from outside HCFs (Table 2). The proportion of patients with comorbidities such as malignancy, diabetes, dementia, and congestive heart failure increased as well. The mean age of inpatients increased from 61.7 to 63.3 years over the study period, and those aged ≥ 70 years accounted for 39.6% of all inpatients in phase 2 (Supplementary Table 1). The mean age of patients who were transferred from outside HCFs was higher than those who were not (72.4 vs. 62.2 years, *P* < 0.001). During phase 1 and 2, 13,962 patients were screened for indications (2,149 in phase 1 and 11,813 in phase 2), and 1,851 patients for other purposes. The proportion of admitted patients meeting screening criteria increased from 14.6% (n = 3,250) in Phase 1 to 44.4% (n = 12,100) in Phase 2. Over the study period, the monthly compliance with the CPE screening program markedly increased from 18.3 to 93.5% (Fig. 1). On average, compliance with the screening program significantly increased between phase 1 and 2, from 59.3 to 86.3% (*P* < 0.001). Weekly ICU screening was performed in 83.7% of the ICU-admitted patients (640/765).

**Table 2:** Comparisons of patient eligible for screenings, new CPE patients by sample and screen, and the incidence of CPE colonization or infection before (Phase 1) and after (Phase 2) the enhanced screening program

	Phase 1	Phase 2		*P*-value
Number of patients with risk factors (%)				
Previous colonization/infection	12 (0.05)	111 (0.4)		
Previous admission to HCFs within 6 months	6,662 (30.0)	8,889 (32.6)		< 0.001
Receipt of hemodialysis	620 (2.8)	798 (2.9)		< 0.001
Transfer from outside HCFs	1406 (6.3)	2111 (7.7)		< 0.001
Number of ICU patients eligible for screening (%)				
ICU universal admission screening	2,376 (10.7)	2,652 (9.7)		< 0.001
ICU weekly screening	NA	765 (2.8)		
Positive screening per 1,000 screens^1^			IRR (95% CI)	*P*-value
Admission screening	11.7	5.3	0.45 (0.29–0.71)	0.001
ICU universal admission screening	9.4	4.4	0.47 (0.19–1.14)	0.07
ICU weekly screening		18.8		
Positive screening per 1,000 admissions^1^			IRR (95% CI)	*P*-value
Admission screening	1.2	2.3	1.90 (1.21–2.99)	0.005
ICU universal admission screening	5.5	4.2	0.76 (0.31–1.83)	0.507
ICU weekly screening		15.7		
Incidence of CPE colonization/infection per 1,000 admissions			IRR (95% CI)	*P*-value
Total new CPE patients	3.1	3.4	1.08 (0.79–1.48)	0.613
New CPE from screening samples	2.6	3.3	1.27 (0.91–1.76)	0.162
New CPE from clinical samples	0.5	0.1	0.20 (0.06–0.72)	0.014
Hospital-onset CPE patients	1.9	1.1	0.57 (0.36–0.91)	0.018
Patients with CPE-positive clinical cultures^1^	0.7	0.4	0.65 (0.31–1.39)	0.270

Abbreviations: CI, confidence interval; CPE, carbapenemase-producing Enterobacterales; ICU, intensive care unit; IRR, incidence rate ratio; HCF, healthcare facility; NA, not applicable

Footnote: ^1^ Patients with CPE isolates detected in clinical samples before or after the identification of CPE colonization through screening, or in cases where CPE screening was not conducted, were included

### Trends in CPE colonization/infection and epidemiological characteristics of CPE cases

The annual incidence of CPE-colonization/infections substantially increased from 0.10 to 4.2 per 1,000 admissions over the 4-year study period. A total of 167 CPE cases were newly identified during the study period, including 149 cases from surveillance testing and 18 cases from clinical cultures. One patient who was previously colonized with NDM-1-producing *Klebsiella oxytoca* was found to carry a different CPE isolate (KPC-2-producing *Klebsiella pneumoniae*). A total of 24 CPE cases were identified through universal ICU admission screening, and 10 of them (41.7%) had no indications other than ICU admission. There were 30 patients with clinical cultures positive for CPE (3 in phase 0, 15 in phase 1, and 12 in phase 2), the most common site being urine (n = 16), followed by sputum (n = 8), wounds (n = 3), and blood (n = 3).

In terms of epidemiologic categorization, there were three HO cases and one CO-HA case during phase 0. During phases 1 and 2, there were 47 CO-HA patients (42 associated with this hospital and five associated with other facilities) and 39 HO-OHCF patients, including 21 from ACHs and 18 from LTCFs or nursing homes. Four CPE cases were considered to be community-associated. There were 73 HO cases associated with this hospital, including 29 cases detected during the outbreak period (Fig. 1).

**Fig. 2:**
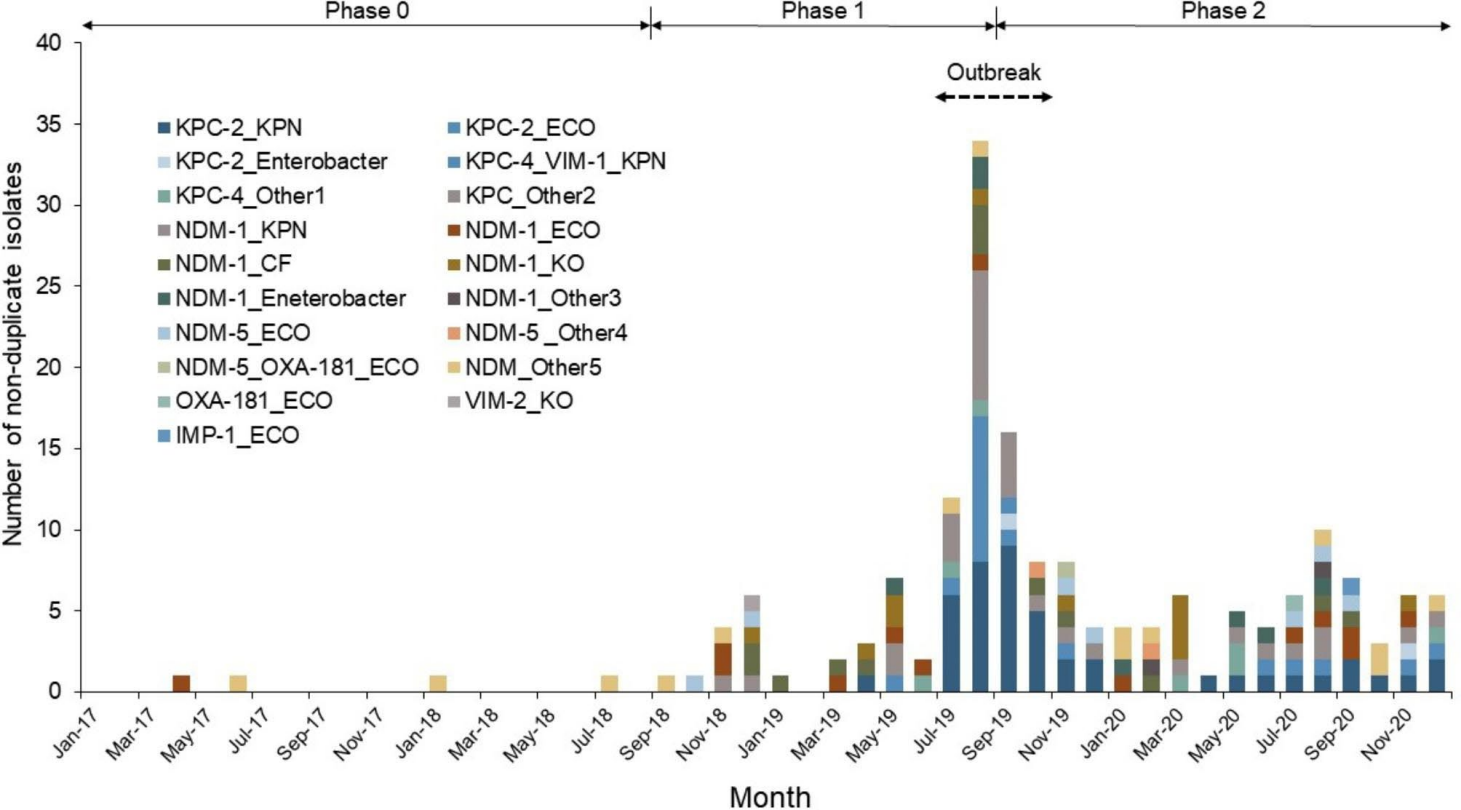
Trend in non-duplicate CPE isolates by bacterial species and carbapenemase genes (2017–2020) Abbreviations: CPE, carbapenemase-producing Enterobacterales; KPN, *Klebsiella pneumoniae*; ECO, *Escherichia coli*; CF, *Citrobacter freundii*; KO, *Klebsiella oxytoca*. Footnote: Other Enterobacterales are as follows: other^1^ includes *Citrobacter, E. coli, K. oxytoca, and K. pneumoniae*; other^2^ includes *K. pneumoniae* and *E. coli*; other^3^ includes *Citrobacter* and *K. aerogenes*; other^4^ includes *C. freundii* and *K. pneumoniae*; other^5^ includes *K. pneumoniae, E. coli*, and *E. asburiae*

In terms of CPE isolates, there were 175 non-duplicate CPE isolates identified, including 8 non-duplicate isolates from 4 patients. Species of CPE isolates and carbapenemase genes were diverse throughout the study period (Fig. 2). Nonetheless, 47.4% (n = 83) of cultures were *K. pneumoniae*, and NDM-1 (39.4%, 69/175) and KPC-2 (31.4%, 55/175) were the most commonly identified carbapenemases. These genes were also frequently detected in patients who were transferred from outside HCFs with NDM-1 accounting for 33.3% (13/39) and KPC-2 accounting for 35.9% (14/39).

### Impact of the enhanced screening program

Compared to phase 1, the incidence of CPE increased from 1.2 to 1,000 admissions to 2.3 per 1,000 admissions in phase 2. The total number of patients eligible for admission screening increased from 3,250 in phase 1 to 12,100 in phase 2, and the proportion of positive admission screens decreased during phase 2 (Table 2). Although the incidence of newly detected cases was higher during phase 2, a significant decrease was observed in the incidence of newly detected CPE cases from clinical samples without a preceding positive screening, from 0.54 to 1,000 admissions to 0.11 per 1,000 admissions (incidence rate ratio [IRR] 0.20; 95% confidence interval [CI] 0.06–0.72; *P* = 0.014) (Table 2), and CPE-positive clinical cultures per 1000 admissions did not change significantly (IRR 0.65; 95% CI 0.31–1.39; *P* = 0.27). The incidence of HO cases also decreased during phase 2 (IRR 0.57; 95% CI 0.36–0.91; *P* = 0.018) (Table 3).

**Table 3:** The relative risk of factors associated with CPE-positive admission screening during the study period after the initiation of screening program (September 2018-December 2020)

	Proportion of CPE positive screening (%)	RR (95% CI, *P*-value)	aRR (95% CI, *P*-value)
Previous colonization/infection			
No	90 (0.6)		
Yes	39 (31.7)	53.30 (38.36–74.07, *P* < 0.001)	37.25 (25.68–54.04, *P <* 0.001)
Transfer from acute care hospitals			
No	108 (0.9)		
Yes	21 (1.3)	1.52 (0.95–2.42, *P* = 0.078)	3.09 (1.93–4.95, *P <* 0.001)
Transfer from long-term care facilities			
No	107 (0.8)		
Yes	22 (1.8)	2.15 (1.36–3.39, *P =* 0.001)	1.66 (1.05–2.61, *P =* 0.029)
Previous hospitalization within 6 months			
No	29 (0.5)		
Yes	100 (1.2)	2.53 (1.67–3.82, *P* < 0.001)	2.30 (1.44–3.68, *P =* 0.001)
Receipt of hemodialysis			
No	109 (0.8)		
Yes	20 (2.6)	3.18 (1.98–5.09, *P* < 0.001)	1.68 (1.09–2.60, *P =* 0.020)

Abbreviations: CI, confidence interval; CPE, carbapenemase-producing Enterobacterales; RR, relative risk; aRR, adjusted relative risk

Footnote: ^1^Adjusted for risk factors above, age and sex

Overall, 42 additional patients were identified during phase 2 by extending the admission screening indications (n = 30) and weekly in-ICU screening (n = 12). These accounted for 45.2% (42/93) of the newly detected CPE cases during phase 2.

In comparison to phase 1, the median exposure duration to CPE was significantly reduced during phase 2, from 10.8 days (interquartile range [IQR] 2.6–21.0) to 1 day (IQR 0.5–2.6) (*P* < 0.001). The median number of CPE contacts was also reduced during phase 2, from 11 contacts (IQR 4–19) to 1 contact (IQR 0–5) (*P* < 0.001). During phase 2, no widespread outbreaks occurred, although there were small clusters or sporadic CPE cases.

When analyzing the pre-outbreak, outbreak and post-outbreak periods, similar findings were observed. Compared to the pre-outbreak period, a significant reduction was observed in the incidence of newly detected CPE cases from clinical samples (0.5 vs. 0.1 per 1,000 admissions, *P* = 0.033), the exposure duration (7.6 days vs. 1.0 days, *P* < 0.001), and the number of CPE contacts (9.0 vs. 1.0, *P* < 0.001) during the post-outbreak period. The incidence of HO cases was similar between the pre-and post-outbreak period (0.8 vs. 0.9; *P* = 0.528) despite a substantial increase in overall CPE incidence (1.4 vs. 3.1 per 1,000 admissions, *P* < 0.001) (Supplementary Table 2).

### Risk associated with CPE colonization at admission

The risks of CPE-positive admission screening are summarized in Table 3. The risk of CPE positive admission screening was the highest in patients with previous CPE colonization/infection with the proportion of positive screening upon readmission being 31.7% (39/123) (adjusted relative risk [aRR] 37.25, 95% confidence interval [CI] 25.68–54.04; *P* < 0.001). Among patients without previous CPE colonization/infection, the risk of having a positive CPE screening was highest among patients transferred form ACHs or LTCFs. Also, receipt of hemodialysis was identified as an independent risk factor with the proportion of CPE positive admission screening being 1.7% (13/784) (Supplementary Table 3). The risk of CPE screening positivity did not differ between patients receiving HD at other hospitals compared to those receiving HD at this hospital (RR 1.10, 95%CI 0.36–3.32, *P* = 0.869).

## Discussion

This study showed that previously unrecognized CPE carriers were detected using the enhanced screening program. More than 45% of CPE cases were additionally detected through the enhanced screening program and increased compliance. Application of the Xpert Carba-R assay in combination with screening cultures reduced the exposure duration and number of close contacts in hospital settings. These factors may have prevented widespread CPE outbreaks and reduced the incidence of CPE clinical infections. Universal screening of ICU patients at admission and weekly thereafter was useful for detecting CPE colonization early and potentially reducing the risk of clinical infections in critically ill patients. In particular, weekly ICU screening is considered as an important component of the surveillance program as the positivity rate among patients undergoing weekly ICU screening was higher than those undergoing ICU admission screening.

In South Korea, the prevalence of CRE has increased rapidly since 2017, and CPE constitutes 73.9% of the CRE collected from 2017 to 2020 [12]. Among them, KPC-2 was the predominant carbapenemase (KPC-2 73.8%), followed by NDM-1 (12.9%) and OXA-181 (1.8%). KPC-2-producing *K. pneumoniae* accounted for 58.7% of CPE [12]. This increasing trend correlates with the findings of this study, and the high prevalence of bacteria with the KPC-2 and NDM-1 genes in our hospital reflects the nationwide spread of KPC-2- or NDM-1-carrying Enterobacterales in South Korea. However, in this study, the prevalence of NDM-1 (39.4%) was higher than that of KPC-2 (31.4%) despite KPC-2 being more commonly identified at the national level. This finding indicates the possibility of intra-hospital transmission of NDM-1 carrying Enterobacterales in this hospital.

Among the CRE cases reported to the KDCA, > 90% were colonized cases [13], suggesting that the number of healthcare facilities performing active surveillance increased from 2017 to 2021, as did the number of CPE-colonized patients. In studies conducted before 2017, none of the screened patients were positive for CPE [14], whereas after 2017, 1.4–1.8% of screened patients were CPE-positive at the time of admission [15]. In our study, the proportion of CPE positivity among the screened patients was lower than that reported in previous studies because of the extended indications for CPE screening. However, the incidence of patients positive for CPE screening per 1000 admissions increased from 1.2 in phase 1 to 2.3 in phase 2, which indicates an increasing risk of carrying CPE among inpatients in South Korea and highlights the importance of active CPE screening.

In this study, the mean age of inpatients at this hospital steadily increased over the study period. This aging demographics of the inpatients at this hospital may also have influenced the high detection rate at admission screening during phase 2. As South Korea is an aging society, the number of elderly patients staying at long-term care hospitals or nursing homes has increased [16]. The movement of elderly patients between ACHs and LTCFs may increase the risk of acquisition of CPE. In a study examining CPE acquisition rates in LTCFs in South Korea, the positivity rate was 22.5% for patients sharing a room with a CPE-positive patient [17]. In LTCFs, patients are at an increased risk of persistent colonization due to frequent admissions and readmissions to ACHs, comorbidities, and dependency on nursing care [18]. Transfer from ACHs also poses a high risk of CPE colonization [18–20]. The findings of this study provide additional evidence that patients transferred from both LTCFs and ACHs are at increased risk of CPE carriage compared to those from the community. However, not all healthcare facilities perform CPE screening, and the CPE status of transferred patients is not always communicated. Thus, CPE screening is required to verify CPE colonization status in patients transferred from outside HCFs, which possibly poses the risk of covert transmission before detection. Interfacility patient transfer plays an important role in the spread of CPE [21] and clonal spread of CPE within a region and across the country has been identified in South Korea [12, 22]. Thus, active screening is recommended for patients transferred from either LTCFs or ACH, and interfacility communication should be improved for the timely identification of CPE-colonized patients [23].

As CPE endemicity increases and population demographics change, the risk factors for CPE colonization can broaden. CPE acquisition is more likely to be caused by within-hospital transmission and interfacility spread within the same country rather than by international travel [21, 24]. Consistent with the evolving risk factors, this study also showed considerable intra-hospital transmission. The CPE positivity among the weekly screened ICU patients was higher than that of patients screened upon ICU admission and that HO CPE colonization/infection continued to occur in the post-outbreak period, despite the implementation of the enhanced admission screening program in Phase 2. In our hospital, the initial CPE screening program with narrow indications failed to effectively detect CPE-colonized patients, which allowed an undetected influx of CPE patients into the hospital and subsequent dissemination of CPE. In this study, the prevalence of newly detected CPE colonization among dialysis patients was relatively high at the time of admission (1.7%). Although the prevalence of CPE among dialysis patients has not been widely studied, hemodialysis patients are considered at risk of CPE colonization as they are repeatedly exposed to healthcare settings [3, 18, 25]. One study in Korea showed that approximately 8.4% (14/165) carried CRE and 85.7% (12/14) of those isolates harbored KPC-type carbapenemase [26]. In South Korea, the number of dialysis patients has increased annually, reaching 108,873 patients in 2019, more than 50% of whom were over 65 years of age [27]. Therefore, appropriate infection prevention measures should be implemented in hemodialysis units, and the risk of CPE colonization among dialysis patients should be studied further.

The ICU is a high-risk unit for CPE transmission with a detrimental impact on clinical outcomes. Thus, most guidelines recommend the active screening of ICU-admitted patients. However, screening strategies for ICU patients vary, including universal or targeted screening with or without periodic follow-up screening. Also, the CPE positivity among ICU patients vary depending on the screening strategies. Previous studies reported that the CPE positivity among ICU patients ranged from 0.6% using universal screening to 7.5% using targeted screening for patients transferred from outside facilities [23, 28–31]. From the perspective of test efficiency, universal screening may be labor-intensive and less cost effective-, but many patients would be missed if targeted screening was used [32]. Our study showed a relatively low CPE positivity (0.48%) among ICU patients who underwent universal screening. However, it is notable that a substantial proportion of CPE-positive ICU patients (41.7%) were solely identified through universal screening. As the acquisition of CPE is frequent during the ICU stay, periodic CPE screening among ICU patients is important for detecting patients with CPE colonization and prompting timely infection prevention and control (IPC) measures. In our study, 12 patients with CPE were discovered through weekly screening, which potentially contributed to preventing widespread CPE dissemination in ICUs.

Potential barriers to active CPE screening include a shortage of single rooms, limited availability of screening tests, and a lack of leadership and government efforts to contain CPE [33]. Even with the implementation of active screening, it is challenging to preemptively isolate patients in healthcare settings with a shortage of single rooms. Therefore, in such healthcare settings, as an alternative to preemptive isolation, rapid detection with the Xpert-Carba-R assay can reduce the exposure duration. This study showed that the exposure duration and number of patients exposed to CPE cases were markedly reduced after incorporating the Xpert Carba-R assay for screening. Furthermore, increased awareness of CPE among leaders and HCWs plays a crucial role in the successful implementation of active screening. In our hospital, the importance of CPE prevention was widely recognized during the CPE outbreak, and compliance with CPE screening increased over time through collaborative efforts by IPC units, laboratory department, HCWs, and leadership. During the CPE outbreak, the education on CPE management was reinforced among HCWs, and their compliance with infection prevention measures and the screening program was continued to be monitored and communicated even after the outbreak. These concerted efforts led to the significant improvement in compliance between phase 1 and phase 2, which was critical in containing the CPE outbreak.

This study has a few limitations. Due to the retrospective nature of the study, medical histories from other hospitals may have been incomplete. Despite this, our study had high compliance and well-designed screening plans during phase 2. Secondly, cost-effectiveness was not considered. The enhanced screening program led to more testing and higher expenses in terms of contact precaution measures. However, preventing CPE infections and outbreaks could decrease the costs of additional care and longer hospital stays. Cost-effectiveness studies in other countries have concluded that screening is more economical overall than not screening in CPE-prevalent settings with a colonization prevalence of > 0.015% [34, 35]. In South Korea, the estimated prevalence of CPE was approximately 0.029% in 2023, based on the number of CPE cases reported to the KDCA and the estimated Korean population of 51.4 million in the given year [2, 36]. Therefore, CPE screening can be cost-effective in South Korea. Thirdly, the impact of the enhanced screening program itself may be overestimated. The observed significant reduction in HO CPE cases during phase 2, in comparison to phase 1, could have been attributed to suboptimal compliance during phase 1. With increased compliance during phase 1, the incidence of HO CPE cases would have decreased, even in the absence of the additional CPE screening measures.

## Conclusions

This study showed that an enhanced CPE screening program with high compliance enabled us to identify previously unrecognized CPE-colonized patients in a timely manner and to rapidly institute contact precaution measures that reduced CPE transmission and curtailed a widespread CPE outbreak. As CPE endemicity increases, the risk factors for CPE colonization may broaden, and hospital prevention strategies need to be tailored to changes in regional CPE epidemiology.

## Electronic supplementary material

Below is the link to the electronic supplementary material.

Supplementary Material 1

## Data Availability

Not applicable.

## Abbreviations

ACH: acute care hospital
CA: community-associated
CO-HA: community-onset, healthcare-associated
CPE: Carbapenemase-producing Enterobacterales
CRE: carbapenem-resistant Enterobacterales
HCF: healthcare facility
HO: hospital-onset
HO-OHCF: healthcare-onset, outside healthcare facility
ICU: intensive care unit
IQR: interquartile range
IRR: incidence rate ratio
KDCA: Korea Disease Control and Prevention Agency
LTCF: long-term care facilities
PCR: polymerase chain reaction
RR: relative risk
