# Supplementary material for: The impact of enhanced screening for carbapenemase-producing Enterobacterales in an acute care hospital in South Korea
Source: Antimicrob Resist Infect Control. 2023 Jul 3;12:62. doi: 10.1186/s13756-023-01270-8 (PMC10318785; doi:10.1186/s13756-023-01270-8)
Supplement: Supplementary file 1 — Supplementary Material 1 [file 13756_2023_1270_MOESM1_ESM.pdf]

Additional File 1.

Supplementary Table 1. Demographics of inpatients by each phase during the entire study period (phase 0, 1, and 2) (2017-2020).

	Phase 0	Phase 1	Phase 2	*P*-value
Characteristics (%)				
Number of admissions	30,858	22,228	27,262	
Mean age ($\pm$ SD), years	61.7 (17.0)	62.4 (16.9)	63.3 (16.5)	<0.001
Age ≥ 70 years	11628 (37.7)	8638 (38.9)	10808 (39.6)	<0.001
Sex				
Female	14881 (48.2)	11354 (51.1)	13794 (50.6)	<0.001
Male	15977 (51.8)	10874 (48.9)	13468 (49.4)	
Comorbid conditions				
Diabetes mellitus	8275 (26.8)	6064 (27.3)	8202 (30.1)	<0.001
Liver cirrhosis	1400 (4.5)	950 (4.3)	1059 (3.9)	<0.001
Malignancy	6079 (19.7)	5101 (22.9)	6599 (24.2)	<0.001
Metastatic malignancy	2520 (8.2)	1,556 (7.0)	1,766 (6.5)	<0.001
Chronic lung diseases	950 (3.1)	732 (3.3)	726 (2.7)	<0.001
Cerebrovascular diseases	1455 (4.7)	1164 (5.2)	1421 (5.2)	0.006
Dementia	2539 (8.2)	2338 (10.5)	2883 (10.6)	<0.001
Peripheral vascular diseases	513 (1.7)	354 (1.6)	444 (1.6)	0.821
Congestive heart disease	974 (3.2)	1253 (5.6)	1405 (5.2)	<0.001
Acute myocardial infarction	327 (1.1)	222 (1.1)	275 (1.0)	0.745
Cardiovascular disease	1378 (4.5)	1113 (5.0)	1059 (3.9)	<0.001
CKD stage 4-5	1259 (4.1)	1097 (4.9)	1319 (4.8)	<0.001
Transplant status	162 (0.5)	146 (0.7)	164 (0.6)	0.136
Number of patients with risk factors & ICU admission episodes (%)				
Previous colonization/infection	0 (0.0)	12 (0.05)	111 (0.4)	<0.001
Previous hospitalization within 6 months	8209 (26.6)	6,662 (30.0)	8,889 (32.6)	<0.001
Receipt of HD	647 (2.1)	620 (2.8)	798 (2.9)	<0.001
Transfer from other HCFs	1381 (4.5)	1406 (6.3)	2111 (7.7)	<0.001
ICU admission from ED	1803 (5.8)	1760 (7.9)	1807 (6.6)	<0.001
ICU admission from GW	589 (1.9)	609 (2.7)	845 (3.1)	<0.001

Abbreviation: SD, standard deviation; CKD, chronic kidney disease; HD, hemodialysis; HCF, healthcare facility; ICU, intensive care unit; ED, emergency department; GW, general ward

Supplementary Table 2. The Incidence of CPE colonization or infection according to pre-outbreak (September 2018-June 2019), outbreak (July-September 2019) and post-outbreak period (October 2019-December 2020).

	Incidence (per 1000 admissions)	IRR (95% CI)	*P*-value
Total newly detected CPE patients			
Pre-outbreak	1.4		
Outbreak	10.1	7.35 (4.61-11.74)	<0.001
Post-outbreak	3.1	2.28 (1.45-3.57)	<0.001
New CPE from screening samples			
Pre-outbreak	0.9		
Outbreak	9.5	10.90 (6.26-19.01)	<0.001
Post-outbreak	3.0	3.43 (1.99-5.87)	<0.001
New CPE from clinical samples			
Pre-outbreak	0.5		
Outbreak	0.5	1.04 (0.28-3.84)	0.955
Post-outbreak	0.1	0.24 (0.07-0.89)	0.033
Hospital-onset CPE patients			
Pre-outbreak	0.8		
Outbreak	6.0	7.79 (4.19-14.48)	<0.001
Post-outbreak	0.9	1.24 (0.64-2.39)	0.528
Patients with CPE-positive clinical cultures[1]			
Pre-outbreak	0.5	Reference	
Outbreak	1.4	2.77 (1.07-7.18)	0.036
Post-outbreak	0.4	0.80 (0.33-1.97)	0.630

Abbreviations: CI, confidence interval; CPE, carbapeneamse-producing Enterobacterales; IRR, incidence rate ratio

Footnote: [1] Patients with CPE isolates in clinical specimens before or after the identification of CPE colonization through screening, or in cases where CPE screening was not conducted, were included.

Supplementary Table 3. The relative risk of factors associated with CPE positive admission screening among patients without previous CPE colonization or infection.

Factors	Proportion of CPE positive screening (%)	RR (95% CI, *P*-value)	aRR[1] (95% CI, *P*-value)
Transfer from acute care hospitals			
No	69 (0.6)	-	-
Yes	21 (1.3)	2.36 (1.44-3.82, *P*=0.001)	5.25 (2.96-9.29, *P*<0.001)
Transfer from long-term care facilities			
No	72 (0.6)	-	-
Yes	18 (1.4)	2.59 (1.55-4.33, *P*<0.001)	3.21 (1.70-6.04, *P<0.001*)
Previous hospitalization within 6 months			
No	27 (0.5)	-	-
Yes	63 (0.8)	1.73 (1.10-2.71, *P=0.017*)	3.08 (1.84-5.15, *P*<0.001)
Receipt of hemodialysis			
No	77 (0.6)	-	-
Yes	13 (1.7)	2.98 (1.64-5.19, *P*<0.001)	2.54 (1.40-4.61, *P*=0.002)

Abbreviations: CPE, carbapenemase-producing Enterobacterales; RR, relative risk; aRR, adjusted relative risk.

Footnote: [1]Adjusted for risk factors above, age and sex, dementia.
